# Multiwalled carbon nanotubes inhibit cell migration and invasion by destroying actin cytoskeleton via mitochondrial dysfunction in ovarian cancer cells

**DOI:** 10.18632/aging.104130

**Published:** 2020-12-03

**Authors:** Ping Zhang, Jiangyan Teng, Lijuan Wang

**Affiliations:** 1 Department of Reproductive Medicine, Linyi People's Hospital, Linyi, Shandong, China; 2 Department of Tuberculosis, Linyi People's Hospital, Linyi, Shandong, China; 3 Supervision of Medical Areas, Linyi People's Hospital, Linyi, Shandong, China

**Keywords:** multiwalled carbon nanotubes, cytotoxicity, tumor metastasis, actin cytoskeleton, mitochondrial function

## Abstract

Objective: This study aimed to investigate the effects of multiwalled carbon nanotubes (MWCNTs) on cytotoxicity and tumor metastasis in ovarian cancer cells, and further explored its mechanism.

Results: MWCNTs significantly inhibited cell viability and the clone number, increased the cell number of S phage, promoted cell apoptosis, as well as suppressed cell migration and invasion, and damaged the structure of actin cytoskeleton in a dose-dependent manner in SKOV3. Moreover, MWCNTs treatment obviously damaged the structure of actin cytoskeleton of SKOV3, and inhibited the activities of mitochondrial electron transfer chain complexes I-V.

Conclusions: MWCNTs might influence the assembly of actin cytoskeleton by disrupting mitochondrial function, thereby inhibiting migration and invasion of SKOV3.

Methods: The characterization of MWCNTs was analyzed by UV visible light absorption spectroscopy and transmission electron microscopy. SKOV3 cells were exposed to different doses of MWCNTs. Then, *in vitro* cytotoxicity of MWCNTs was evaluated by MTT assay, colony-forming assay, cell cycle, and cell apoptosis assay. Moreover, the effects of MWCNTs on cell migration and invasion as well as actin cytoskeleton were explored in SKOV3 cells. Furthermore, the mitochondrial membrane potential and the activities of mitochondrial electron transfer chain complexes I-V were measured.

## INTRODUCTION

As one of the most common gynecological malignancies, ovarian cancer is a highly fatal female cancer [1]. Ovarian cancer statistics estimate that approximately 52,100 newly diagnosed ovarian cancer and 22,500 death caused by ovarian cancer are reported in 2015 in China [2]. Early diagnosis and effective treatment are effective methods to prevent further deterioration of ovarian cancer [3]. Nowadays, studies have reported the intensive progress in the diagnosis and treatment of ovarian cancer, which contributes to the decreasing mortality and morbidity worldwide [4, 5]. Unfortunately, despite surgery and radiotherapy are effective therapeutic methods for early-stage ovarian cancer, poor prognosis still exists in advanced ovarian cancer due to high metastasis rate [4, 5]. Therefore, it is essential to further reveal the etiology and tumorigenesis of ovarian cancer and search for novel treatment methods associated with tumor aggressiveness.

Currently, nanomaterials have been reported to be promising in cancer detection and treatment, such as imaging [6], immunodetection [7], chemotherapy [8], radiotherapy [9], and immunotherapy [10]. Carbon nanotubes (CNTs) are one class of highly attractive nanomaterials due to exceptional physicochemical properties, including low weight, high flexibility, resistance against corrosion, special electrical performance and excellent mechanical strength [11]. CNTs can result in many adverse effects in living organisms by penetrating into cells and tissues [12–14]. Particularly, multiwalled carbon nanotubes (MWCNTs) have attracted tremendous attention and displayed great application values in various fields, such as materials, information, energy and biomedicine [15]. Based on the unique mechanical strength of MWCNTs, they can be adsorbed on the surface of cell membranes to affect cell membrane stiffness and fluidity, thereby changing the biomechanical properties of cells [16]. The cell biomechanical characteristics highly influence the cell movement process by involving with cytoskeleton remodeling and cell membrane flow [16]. Several studies have proved the anti-proliferative and pro-apoptotic effects of MWCNTs through influencing microtubule function of cells [17, 18]. Moreover, only a few studies have claimed that MWCNTs may inhibit cancer cell migration [19, 20]. However, the effect and mechanism of MWCNTs in ovarian cancer has not been further investigated.

In the current study, the effects of MWCNTs on cytotoxicity and tumor metastasis were unraveled in ovarian cancer SKOV3 cells, and the potential mechanism was further explored.

## RESULTS

### Characterization of MWCNT

**Figure 1:**
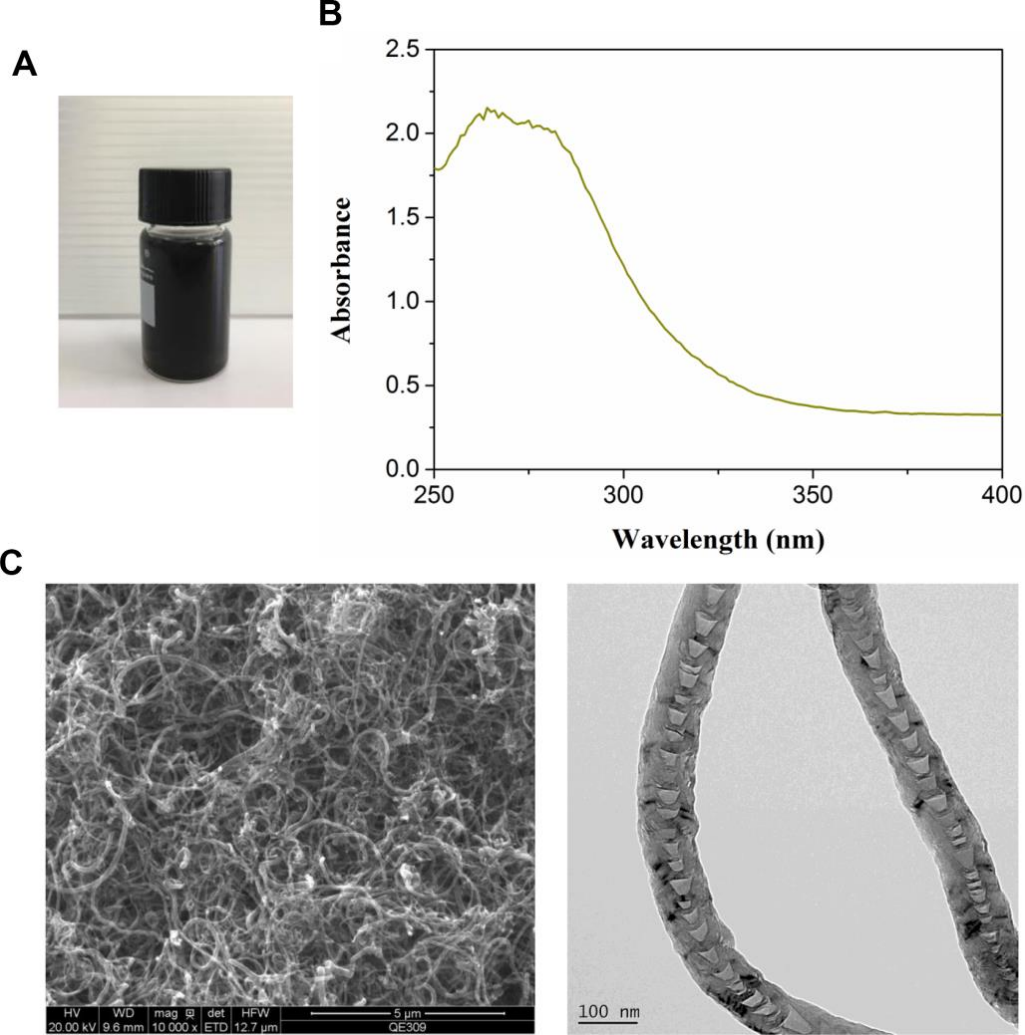
**Characterization of multiwalled carbon nanotubes (MWCNTs).** (**A**) The appearance of commercial MWCNTs. (**B**) The ultraviolet visible absorption spectra of MWCNTs. (**C**) The representative images of MWCNTs using transmission electron microscopy.

Commercial MWCNT dispersion exhibited black appearance (Figure 1A). The optical absorption spectra of MWCNT revealed around 260 nm of absorption peaks (Figure 1B). TEM images showed the complete and visible tubular structure (Figure 1C).

### Effect of MWCNT on cytotoxicity in SKOV3 cells

**Figure 2:**
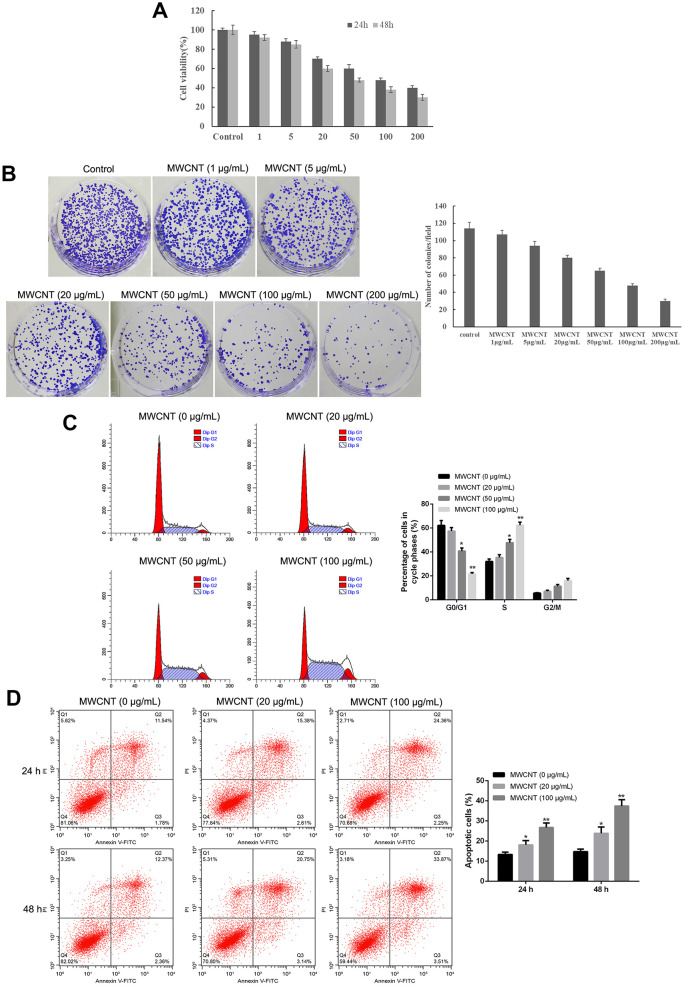
**Multiwalled carbon nanotubes (MWCNTs) inhibits tumor growth in SKOV3 cells.** (**A**) Cell viability of SKOV3 cells treated with different doses of MWCNTs at 24h and 48 h by MTT assay. (**B**) Clone number of SKOV3cells treated with different doses of MWCNTs at 24h by colony formation assay. (**C**) Cell cycle of SKOV3 cells treated with different doses of MWCNTs at 24h by flow cytometry analysis. (**D**) Cell apoptosis rate of SKOV3 cells treated with different doses of MWCNTs at 24h and 48 h by flow cytometry analysis. *P < 0.05, and **P < 0.01 vs control cells (0 μg/mL MWCNTs).

MTT assay showed that MWCNT significantly inhibited cell viability in dose-dependent manner both at 24 and 48 h, and MWCNT with a dose of less than 100 μg/mL showed more than 50% of cell viability (Figure 2A). Another ovarian cancer cell line A2780 and used MTT to detect the effect of different concentrations of MWCNTs on cell viability. Consistently, colony formation results also revealed that compared with control cells, the clone number was obviously decreased in SKOV3 cells with MWCNT in dose-dependent manner (Figure 2B). In addition, flow cytometry analysis found that MWCNT treatment prominently reduced the cell number of G0/G1 phage, while increased cell number of S phage in dose-dependent manner (p < 0.05, Figure 2C). Meanwhile, the rate of apoptotic cells was remarkably increased after MWCNT treatment in dose-dependent manner at 24 h in SKOV3 cells compared with control cells (p < 0.05, Figure 2D).

### Effect of MWCNT on tumor metastasis in SKOV3 cells

**Figure 3:**
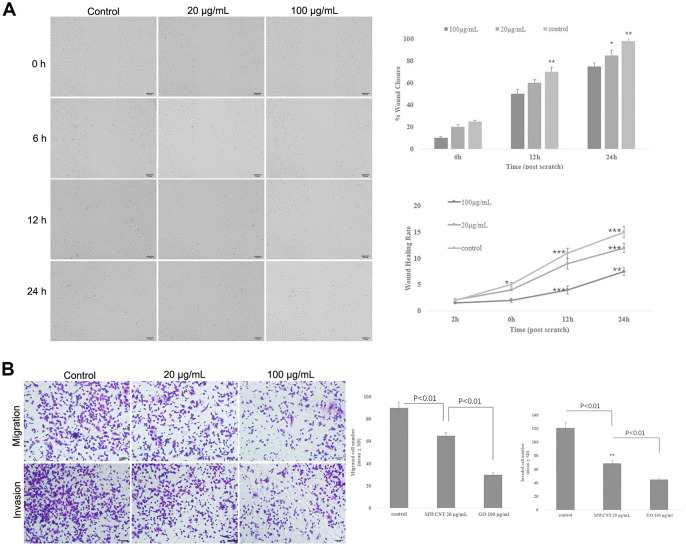
**Multiwalled carbon nanotubes (MWCNTs) inhibits tumor metastasis in SKOV3 cells.** (**A**) The wound closure and wound healing rate of SKOV3 cells treated with different doses of MWCNTs at 0, 6, 12 h and 24 h by wound healing assay. (**B**) Cell migration and migration rates in SKOV3 cells treated with different doses of MWCNTs by Transwell assay. *P < 0.05, **P < 0.01, and ***P < 0.001 vs control cells.

Wound healing assay showed that MWCNT significantly decreased the wound closure and inhibited wound healing rate of SKOV3 cells in dose- and time-dependent manner (p < 0.05, Figure 3A), suggesting a reduced migration potential after MWCNT treatment in SKOV3 cells. Transwell assay also revealed that cell migration and invasion were dramatically inhibited in SKOV3 cells treated with MWCNT compared with control cells (p < 0.05, Figure 3B).

### Effect of MWCNT on actin cytoskeleton of SKOV3 cells

**Figure 4:**
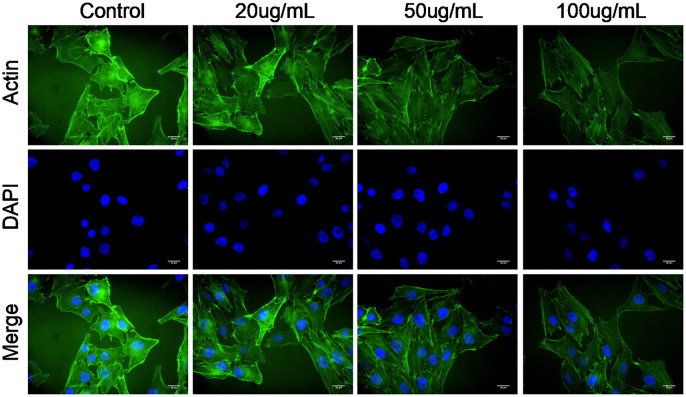
**Multiwalled carbon nanotubes (MWCNTs) disrupts actin cytoskeleton of SKOV3 cells.** Actin cytoskeleton of SKOV3 cells treated with different doses of MWCNTs under confocal microscopy.

Actin cytoskeleton is essential for cell migration and invasion; thus, actin cytoskeleton of SKOV3 cells was observed under confocal microscopy. As shown in Figure 4, the cellular cytoplasm of control cell exhibited the well-arranged actin filaments in thick bundles. In contrast, MWCNT treatment damaged the structure of actin cytoskeleton of SKOV3 cells in dose-dependent manner (Figure 4).

### Effect of MWCNT on mitochondrial function of SKOV3 cells

**Figure 5:**
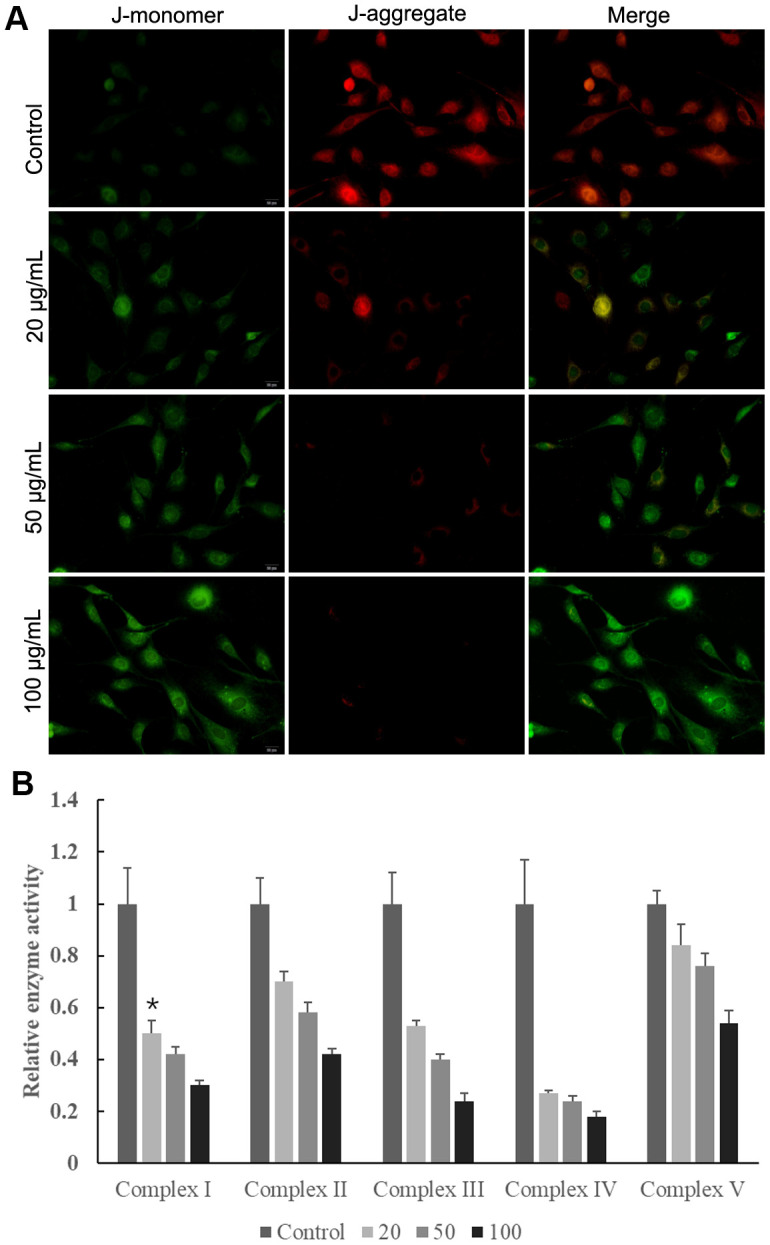
**Multiwalled carbon nanotubes (MWCNTs) disrupted mitochondrial function of SKOV3 cells.** (**A**) The mitochondrial membrane potential in SKOV3 cells treated with different doses of MWCNTs. (**B**) The activities of mitochondrial respiratory chain complexes I-V detected by commercially kits.

The mitochondrial membrane potential results showed that control cells mainly presented JC-1 aggregates (red fluorescence), while increased JC-1 monomers (green fluorescence) and reduced JC-1 aggregates were observed in cells treated with MWCNT in dose-dependent manner (Figure 5A). This indicated that the treatment of MWCNTs caused a decrease in mitochondrial membrane potential. Consistently, when SKOV3 cells exposed to MWCNT for 24 h, the activities of mitochondrial electron transfer chain complexes I-V were significantly decreased in dose-dependent manner compared with un-treated cells (Figure 5B). Here, we detected the level of p-NF-κB and p-p38-MAPK, which related to mitochondrial function. MWCNTs might alter the function of mitochondria by activating MAPK signaling and NF-κB signaling. The OCR detection showed that cells treated with MWCNTs have lower mitochondrial respiration.

## DISCUSSION

The present study revealed that MWCNTs significantly inhibited cell viability and the clone number, increased the cell number of S phage, promoted cell apoptosis, as well as suppressed cell migration and invasion in dose-dependent manner in SKOV3 cells. Moreover, MWCNTs treatment obviously damaged the structure of actin cytoskeleton of SKOV3 cells, and inhibited the activities of mitochondrial electron transfer chain complexes I-V.

MWCNTs have widely used for various biomedical applications due to their unique physiochemical properties, and the biocompatibility and safety of MWCNTs are considered as key factors in biomedical applications. Several studies have evaluated the cytotoxicity of MWCNTs *in vitro* experiments. Graham et al. have reported that MWCNTs with the doses of 0-100 μg/mL exhibit similar cell viability, suggested the minimal cytotoxicity to normal MCF 10A cells as well as breast cancer cells (MDA-231 cells and MCF-7 cells) [20]. Also, García-Hevia L et al. have evaluated the toxicity of MWCNTs in different migrating cancer cells, such as glioma U87MG cells, neuroblastoma SH-SY5Y cells, cervical cancer HeLa cells, and breast cancer MCF-7 cells, and the results show no obvious toxicity when treated with 25 μg/mL of MWCNTs for 70 h [19]. In this study, we also evaluated the cytotoxicity effect of MWCNTs at different doses in SKOV3 cells. The results found that MWCNTs exhibited inhibiting effect on cell viability and the clone number in a dose-dependent manner, while more than 100 μg/mL of MWCNTs showed a low cell viability at 48 h in SKOV3 cells. Furthermore, SKOV3 cells treated with MWCNTs showed a low apoptotic rate at 20 μg/mL of MWCNTs, while S phase arrest and a high apoptotic rate were found at 100 μg/mL of MWCNTs. All these data suggested that MWCNTs with a dose of less than 100 μg/mL exhibited anti-proliferative and pro-apoptotic effects, while non-half lethal cytotoxicity effect in SKOV3 cells.

Tumor metastasis is a complex and multistep process, which includes the formation of a new microvascular network, tumor cells invading the primary organ, escaping from the primary tumor into the surrounding tissue, arresting and proliferation in a new organ [21]. High rate of tumor metastasis is considered a great challenge for cancer treatment [22]. Interestingly, previous studies have shown that MWCNTs treatment contributes to a slower migration and invasion in several cancer cells [17, 19]. Thus, we further investigated the effect of MWCNTs on cell migration and invasion in SKOV3 cells, and the results found that MWCNTs suppressed migration and invasion of SKOV3 cells. It is well-known that tumor cells undergo morphological and structural changes during metastasis, and actin cytoskeleton plays an important role in cancer progression by driving tumor cell invasion and migration [23–25]. In response to various extracellular factors and signals, a characteristic polarized morphology is firstly observed in the migrating cells [26]. Concretely, the fingerlike protrusions (filopodia) and flat membrane protrusions (lamellipodia) are extended at the cell front by driving actin assembly [26]. Then the actin cytoskeleton is connected with the extracellular matrix by cell adhesion at the leading edge of the lamellipodia, thereby leading to cell migration [26]. Importantly, the formation and rearrangement of actin cytoskeleton are induced by activated actin under external stimuli [27, 28]. Thus, this study observed the structural changes of the actin cytoskeleton by actin staining, and the results showed that MWCNTs damaged the structure of actin cytoskeleton of SKOV3 cells. Importantly, energy is necessary for the assembly of the actin cytoskeleton, and intracellular ATP is usually produced from mitochondria in mammalian cells [29]. In addition, the mitochondrial electron transfer chain complexes I-V have been suggested to play key roles in ATP synthesis [29]. Thus, mitochondrial function was investigated by the measurements of the mitochondrial membrane potential and the activities of mitochondrial electron transfer chain complexes I-V. Our study revealed that MWCNTs obviously damaged the structure of actin cytoskeleton of SKOV3 cells, and inhibited the activities of mitochondrial electron transfer chain complexes I-V. These data indicated that MWCNTs might influence the assembly of actin cytoskeleton by disrupting mitochondrial function, thereby inhibiting migration and invasion of SKOV3 cells.

In conclusion, findings from this study revealed that MWCNTs exhibit inhibiting effects in cell migration and invasion by destroying actin cytoskeleton, which might be associated with mitochondrial dysfunction.

## MATERIALS AND METHODS

### Characterization of MWCNTs

MWCNTs with > 95% purity were purchased from XFNANO Materials Tech Co. Ltd (Nanjing, Jiangsu, China). MWCNTs were dissolved in 1% DMSO and MWCNTs suspension was prepared by sonication machine. The ultraviolet (UV) visible spectra of MWCNTs was measured by UV visible light absorption spectroscopy (Biochrom, Cambridge, UK). The morphology of MWCNTs was observed by transmission electron microscopy (TEM; HT7700; Hitachi, Japan).

### Cell culture and treatment

Ovarian cancer cell line SKOV3 and A2780 were obtained from Shanghai Obio Technology Co., Ltd, and then maintained in complete DMEM medium (Gibco, Carlsbad, CA, USA) under 37° C and 5% CO_2_. SKOV3 and A2780 cells were exposed to different doses of MWCNT.

### MTT assay

SKOV3 and A2780 cells were grown in 96-well plates, and then underwent different doses of MWCNT (1, 5, 20, 50, 100, and 200 μg/mL). After conventional incubation for 24 and 48 h, each well was added with 10 μl of MTT (5 mg/mL, Sigma) for another 4 h. Afterwards, 100 μL of dimethyl sulfoxide was added. Microplate spectrophotometer was used to evaluate cell viability based on the absorbances at 470 nm.

### Colony-forming assay

SKOV3 cells were grown in 6-well plates at a density of 400 cells/well, and then underwent different dose of MWCNT (1, 5, 20, 50, 100, and 200 μg/mL) for 14 days under standard culture conditions. Then cells were fixed with absolute methanol, and incubated with crystal violet. Ultimately, the number of colonies was counted.

### Flow cytometry assay

Cell cycle and apoptosis were evaluated by flow cytometry assay. SKOV3 cells were treated with different doses of MWCNT (20, 50, and 100 μg/mL) for 24 h. Trypsin was used to digest cells with various treatments, and then cells were harvested. For cell cycle assay, cells were incubated in 70% ethanol on ice for 2 h, and then stained with propidium iodide (PI) for 30 min at 37° C. For cell apoptosis, FITC-Annexin V Apoptosis kit was used. Briefly, cells were rinsed with PBS, and resuspended with Binding Buffer. Next, the cells underwent the incubation of FITC-Annexin V and PI with cells for 15 min, in turn. Flow cytometer (BD, CA, USA) was used to measure cell cycle and calculate the number of apoptotic cells.

### Wound healing assay

SKOV3 cells were inoculated in 6-well plates. After growing to 60% of confluence, cells were wounded by scratching the vertical lineation using pipette tips, followed by culturing in DMEM without serum. Subsequently, cells were treated with 0, 20 and 100 μg/mL of MWCNT, respectively. The distance of the scratch was observed at 0, 6, 12 and 24 h using a light microscope (Olympus, Japan).

### Transwell assay

Tumor cell invasion and migration were elevated by Transwell chambers (Corning). Concretely, the bottom compartment was added with DMEM supplemented with 10% FBS and MWCNT (20 and 100 μg/mL). SKOV3 cells were grown in the upper compartment coated with Matrigel Matrix and cultured in medium free of serum for 24 h. Subsequently, the cells in the bottom compartment were fixed and stained 4,6-diamidino-2-phenylindole. An inverted microscope (Olympus, Japan) was utilized to evaluate cell migration and invasion.

### Cytoskeleton staining

SKOV3 cells were inoculated on cover slips, and then exposed to 0, 20, 50 and 100 μg/mL of MWCNT, respectively, for 24 h. The cells were then subjected to fixation using 4% paraformaldehyde and permeabilized treatment by 0.1% Triton X-100. Followed by blockage by 2% bovine serum albumin, the cells were stained with FITC-Phalloidin (actin, green) and DAPI (nucleus, blue), in turn. Finally, cell images were obtained using laser scanning confocal microscope (Olympus, Japan).

### Mitochondrial membrane potential detection

Mitochondrial membrane potential depolarization was detected using commercial JC-1 kit (Beyotime). At high membrane potential, JC-1 shows red fluorescent aggregates; while JC-1 presents green fluorescent monomers at low membrane potential. Briefly, SKOV3 cells were treated with different doses of MWCNT (20, 50, and 100 μg/mL) for 24 h, and then stained with 1 ml of JC-1 dye at 37° C for 20 min. Next, cells were rinsed with JC-1 buffer, and observed under an inverted microscope (Olympus, Japan).

### Activity measurements of mitochondrial respiratory chain complexes

SKOV3 cells were inoculated in 6-well plates, and treated with 0, 20, 50, and 100 μg/mL of MWCNT, respectively, for 24 h. Next, trypsin was used to digest cells with various treatments, and then cells were lysed. Subsequently, the activities of mitochondrial respiratory chain complexes I-V were detected by commercial kits (Jianchen, Nanjing, China).

### Western blot analysis

The SKOV3 cells were lysed using radioimmunoprecipitation assay (RIPA) buffer (Beyotime, Shanghai, China). Total protein levels were quantified using a BCA Protein Assay kit (Pierce; Thermo Fisher Scientifc, Inc.) prior to performing immunoblot analysis. 10% sodium dodecyl sulfate-polyacrylamide gel electrophoresis (SDS-PAGE) was used to separate protein samples (15 mg protein loaded per well) and then proteins were transferred to polyvinylidene difluoride (PVDF) membranes (Millipore, Billerica, MA, USA). The membranes were blocked with 5% non-fat milk in Tris-buffered saline with 0.1% Tween 20 (TBST) at room temperature for 3 h, and then PVDF membranes were incubated with the corresponding primary antibodies (including anti- p-NF-κB, anti-p-p38-MAPK, anti-T**-**p38-MAPK, and anti-β-actin antibodies) at 4° C overnight. The membranes were washed three times with TBST, and then incubated with corresponding secondary antibodies at room temperature for 1 h. Finally, the protein bands were visualized using the enhanced chemiluminescence (ECL) protein detection kit (Millipore, MA, USA). The relative protein expression was analyzed using Image-Pro Plus software version 6.0 (Media Cybernetics, Inc., Rockville, MD, USA) and represented as the density ratio *vs*. β-actin.

### Oxygen consumption rate (OCR) measurement

The Extracellular Flux Analyzer 8-well format (Seahorse Bioscience, Agilent Technologies, USA) was used to synchronously detect the oxygen consumption rate (OCR) in SKOV3 cells.

Briefly, cells were seeded onto XFp cell culture mini plates and cultured overnight. For OCR analysis, 1 μM oligomycin, 1 μM carbonyl cyanide 4-(trifluoromethoxy) phenylhydrazone (FCCP) and 0.5 μM antimycin A (Sigma, USA) were added into special 8-well probe plates. All measurements were performed according to the manufacturer's instructions, and the number of cells in each well was normalized.

### Statistical analysis

Data were presented as the mean ± SD. One-way ANOVA followed by multiple comparison was used for data comparisons based on SPSS software. P < 0.05 was considered significant.
